# Supplementary material for: Evaluation of a Proportional Response Addition Approach to Mixture Risk Assessment and Predictive Toxicology Using Data on Four Trihalomethanes from the U.S. EPA’s Multiple-Purpose Design Study
Source: Toxics. 2024 Mar 25;12(4):240. doi: 10.3390/toxics12040240 (PMC11053411; doi:10.3390/toxics12040240)
Supplement: Supplementary file 1 [file toxics-12-00240-s001.zip › toxics-2904092-supplementary.pdf]

Proportional Response Addition

Supplemental Materials

CONTENTS

Table S1. **Prop-RA Results for Binary Mixture of CHCl3 and BDCM**
Table S2. **Prop-RA Results for Binary Mixture of CHCl3 and BDCM-rep**
Table S3. **Prop-RA Results for Binary Mixture of CHCl3 and CHBr3**
Table S4. **Prop-RA Results for Binary Mixture of BDCM and CHBr3**
Table S5. **Prop-RA Results for Binary Mixture of BDCM and CDBM**
Table S6. **Prop-RA Results for Binary Mixture of BDCM and CDBM-rep**
Table S7. **Prop-RA Results for Binary Mixture of CHCl3 and CDBM**
Table S8. **Prop-RA Results for Binary Mixture of CDBM and CHBr3**
Table S9. **Prop-RA Untransformed Data Results Binary Mixture of CHCl3 and BDCM**
Table S10. **Prop-RA Untransformed Data Results for Binary Mixture of CHCl3 and BDCM-rep**
Table S11. **Prop-RA Untransformed Data Results for Binary Mixture of CHCl3 and CHBr3**
Table S12. **Prop-RA Untransformed Data Results for Binary Mixture of BDCM and CHBr3**
Table S13. **Prop-RA Untransformed Data Results for Binary Mixture of BDCM and CDBM**
Table S14. **Prop-RA Untransformed Data Results for Binary Mixture of BDCM and CDBM-rep**
Table S15. **Prop-RA Untransformed Data Results for Binary Mixture of CHCl3 and CDBM**
Table S16. **Prop-RA Untransformed Data Results for Binary Mixture of CDBM and CHBr3**
Homogeneity of Variances. Prop-RA Test Results for Binary Mixtures

Table S1. Prop-RA Results for Binary Mixture of CHCl3 and BDCM[1,2]

0.5:0.5 CHCl3:BDCM
Dose = 0.1 mmol/kg/day

End Point	CHCl3			BDCM			Mixture			L	Scheffé 95% CI	
	N	Mean	Std Dev	N	Mean	St Dev	N	Mean	St Dev		Low CL	Upp CL
PcLiv	10	4.66	0.48	10	4.72	0.40	11	4.98	0.49	-0.28717	-0.74	0.16
Log(ALT)	10	1.41	0.10	10	1.40	0.11	11	1.40	0.11	0.007	-0.10	0.11
Log(AST)	10	1.61	0.09	10	1.65	0.11	11	1.63	0.11	0.0025	-0.10	0.10
Log(SDH)	10	1.16	0.04	10	1.08	0.16	11	1.16	0.06	-0.0395	-0.14	0.06

0.5:0.5 CHCl3:BDCM
Dose = 1.0 mmol/kg/day

End Point	CHCl3			BDCM			Mixture			L	Scheffé 95% CI	
	N	Mean	Std Dev	N	Mean	St Dev	N	Mean	St Dev		Low CL	Upp CL
PcLiv	9	5.40	0.31	7	6.15	0.83	10	5.76	0.37	0.018606	-0.53	0.56
Log(ALT)	9	1.62	0.13	7	1.91	0.26	11	1.63	0.14	0.13	-0.05	0.31
Log(AST)	9	1.64	0.08	7	1.78	0.17	11	1.63	0.07	0.083	-0.02	0.19
Log(SDH)	9	1.33	0.07	7	1.48	0.23	11	1.37	0.11	0.0325	-0.11	0.18

0.5:0.5 CHCl3:BDCM
Dose = 3.0 mmol/kg/day

End Point	CHCl3			BDCM			Mixture			L	Scheffé 95% CI	
	N	Mean	Std Dev	N	Mean	St Dev	N	Mean	St Dev		Low CL	Upp CL
PcLiv	7	7.72	0.95	7	8.16	1.59	9	8.42	0.93	-0.47626	-1.80	0.85
Log(ALT)	7	2.66	0.42	7	2.91	0.27	9	2.80	0.48	-0.014	-0.48	0.45
Log(AST)	7	2.42	0.46	7	2.85	0.35	9	2.67	0.54	-0.0365	-0.56	0.49
Log(SDH)	7	1.94	0.28	7	1.95	0.41	9	2.05	0.37	-0.1045	-0.51	0.30

[1]Relative Liver Weight calculations use untransformed data. ALT, AST and SDH calculations use Log10 transformed data.

[2]Shaded areas are statistically significant. Overall type I error rate for this study using Scheffé's method is α=0.05.

Table S1 (cont). Prop-RA Results for Binary Mixture of CHCl3 and BDCM[1,2]

0.730:0.270 CHCl3:BDCM
Dose = 1.0 mmol/kg/day

End Point	CHCl3			BDCM			Mixture			L	Scheffé 95% CI	
	N	Mean	Std Dev	N	Mean	St Dev	N	Mean	St Dev		Low CL	Upp CL
PcLiv	9	5.40	0.31	7	6.15	0.83	11	5.68	0.44	-0.07441	-0.63	0.48
Log(ALT)	9	1.62	0.13	7	1.91	0.26	11	1.78	0.17	-0.0847	-0.28	0.11
Log(AST)	9	1.64	0.08	7	1.78	0.17	11	1.81	0.07	-0.13112	-0.24	-0.02
Log(SDH)	9	1.33	0.07	7	1.48	0.23	11	1.40	0.15	-0.03123	-0.20	0.13

0.730:0.270 CHCl3:BDCM
Dose = 3.0 mmol/kg/day

End Point	CHCl3			BDCM			Mixture			L	Scheffé 95% CI	
	N	Mean	Std Dev	N	Mean	St Dev	N	Mean	St Dev		Low CL	Upp CL
PcLiv	7	7.72	0.95	7	8.16	1.59	11	8.11	0.85	-0.26488	-1.51	0.98
Log(ALT)	7	2.66	0.42	7	2.91	0.27	11	2.91	0.40	-0.18388	-0.60	0.23
Log(AST)	7	2.42	0.46	7	2.85	0.35	11	2.72	0.39	-0.18571	-0.63	0.26
Log(SDH)	7	1.94	0.28	7	1.95	0.41	11	2.07	0.27	-0.12465	-0.48	0.23

[1]Relative Liver Weight calculations use untransformed data. ALT, AST and SDH calculations use Log10 transformed data.

[2]Shaded areas are statistically significant. Overall type I error rate for this study using Scheffé's method is α=0.05.

Table S2. Prop-RA Results for Binary Mixture of CHCl3 and BDCM-rep[1,2]

0.5:0.5 CHCl3:BDCM
Dose = 0.1 mmol/kg/day

	CHCl3			BDCM			Mixture				Scheffé 95% CI	
End Point	N	Mean	Std Dev	N	Mean	St Dev	N	Mean	St Dev	L	Low CL	Upp CL
PcLiv	8	5.18	0.39	10	4.91	1.59	10	5.23	0.37	-0.1796	-1.21	0.85
Log(ALT)	8	1.38	0.17	10	1.35	0.14	10	1.39	0.11	-0.027	-0.17	0.12
Log(AST)	8	1.60	0.08	10	1.61	0.11	10	1.58	0.08	0.021	-0.07	0.12
Log(SDH)	8	1.13	0.10	10	1.09	0.07	10	1.12	0.07	-0.0095	-0.09	0.07

0.5:0.5 CHCl3:BDCM
Dose = 1.0 mmol/kg/day

	CHCl3			BDCM			Mixture				Scheffé 95% CI	
End Point	N	Mean	Std Dev	N	Mean	St Dev	N	Mean	St Dev	L	Low CL	Upp CL
PcLiv	9	6.34	0.54	8	6.22	0.50	10	6.30	0.63	-0.01859	-0.61	0.57
Log(ALT)	9	1.70	0.17	7	1.84	0.22	9	1.72	0.26	0.047	-0.19	0.29
Log(AST)	9	1.66	0.05	7	1.76	0.13	9	1.73	0.14	-0.0285	-0.15	0.09
Log(SDH)	9	1.42	0.16	7	1.61	0.18	9	1.48	0.19	0.041	-0.15	0.24

0.5:0.5 CHCl3:BDCM
Dose = 3.0 mmol/kg/day

	CHCl3			BDCM			Mixture				Scheffé 95% CI	
End Point	N	Mean	Std Dev	N	Mean	St Dev	N	Mean	St Dev	L	Low CL	Upp CL
PcLiv	5	8.29	1.78	7	8.41	0.55	9	9.22	1.77	-0.86813	-2.62	0.88
Log(ALT)	3	2.96	0.42	7	2.78	0.18	10	3.02	0.42	-0.154	-0.59	0.29
Log(AST)	3	2.65	0.46	7	2.65	0.16	10	2.81	0.50	-0.16	-0.67	0.35
Log(SDH)	3	2.35	0.44	7	2.23	0.24	10	2.34	0.42	-0.0505	-0.52	0.41

[1]Relative Liver Weight calculations use untransformed data. ALT, AST and SDH calculations use Log10 transformed data.
[2]Shaded areas are statistically significant. Overall type I error rate for this study using Scheffé's method is α=0.05.

Table S2 (cont). Prop-RA Results for Binary Mixture of CHCl3 and BDCM-rep[1,2]

0.730:0.270 CHCl3:BDCM
Dose = 1.0 mmol/kg/day

	CHCl3			BDCM			Mixture				Scheffé 95% CI	
End Point	N	Mean	Std Dev	N	Mean	St Dev	N	Mean	St Dev	L	Low CL	Upp CL
PcLiv	9	6.34	0.54	8	6.22	0.50	10	6.40	0.72	-0.0947	-0.74	0.55
Log(ALT)	9	1.70	0.17	7	1.84	0.22	10	1.63	0.13	0.10842	-0.07	0.29
Log(AST)	9	1.66	0.05	7	1.76	0.13	10	1.65	0.09	0.02827	-0.07	0.13
Log(SDH)	9	1.42	0.16	7	1.61	0.18	10	1.36	0.13	0.1133	-0.05	0.28

0.730:0.270 CHCl3:BDCM
Dose = 3.0 mmol/kg/day

	CHCl3			BDCM			Mixture				Scheffé 95% CI	
End Point	N	Mean	Std Dev	N	Mean	St Dev	N	Mean	St Dev	L	Low CL	Upp CL
PcLiv	5	8.29	1.78	7	8.41	0.55	7	8.43	0.98	-0.11181	-1.66	1.43
Log(ALT)	3	2.96	0.42	7	2.78	0.18	7	2.74	0.46	0.1634	-0.40	0.73
Log(AST)	3	2.65	0.46	7	2.65	0.16	7	2.58	0.52	0.067	-0.56	0.69
Log(SDH)	3	2.35	0.44	7	2.23	0.24	7	2.21	0.42	0.10433	-0.46	0.67

[1]Relative Liver Weight calculations use untransformed data. ALT, AST and SDH calculations use Log10 transformed data.

[2]Shaded areas are statistically significant. Overall type I error rate for this study using Scheffé's method is α=0.05.

Table S3. Prop-RA Results for Binary Mixture of CHCl3 and CHBr3[1,2]

0.5:0.5 CHCl3:CHBr3
Dose = 0.1 mmol/kg/day

	CHCl3			CHBr3			Mixture				Scheffé 95% CI	
End Point	N	Mean	Std Dev	N	Mean	St Dev	N	Mean	St Dev	L	Low CL	Upp CL
PcLiv	10	4.97	0.62	9	5.06	0.33	11	4.75	0.40	0.268194	-0.19	0.73
Log(ALT)	10	1.41	0.14	9	1.46	0.08	10	1.44	0.14	-0.0045	-0.13	0.12
Log(AST)	10	1.63	0.10	9	1.64	0.11	10	1.62	0.08	0.012	-0.09	0.11
Log(SDH)	9	1.28	0.08	9	1.30	0.07	10	1.31	0.08	-0.0265	-0.11	0.05

0.5:0.5 CHCl3:CHBr3
Dose = 1.0 mmol/kg/day

	CHCl3			CHBr3			Mixture				Scheffé 95% CI	
End Point	N	Mean	Std Dev	N	Mean	St Dev	N	Mean	St Dev	L	Low CL	Upp CL
PcLiv	9	5.70	0.56	9	6.42	0.63	9	6.08	0.39	-0.01351	-0.58	0.56
Log(ALT)	9	1.78	0.17	9	1.68	0.21	9	1.68	0.22	0.055	-0.16	0.27
Log(AST)	9	1.67	0.11	9	1.73	0.10	9	1.70	0.14	0.001	-0.12	0.12
Log(SDH)	9	1.63	0.13	9	1.57	0.15	9	1.53	0.08	0.075	-0.06	0.21

0.5:0.5 CHCl3:CHBr3
Dose = 3.0 mmol/kg/day

	CHCl3			CHBr3			Mixture				Scheffé 95% CI	
End Point	N	Mean	Std Dev	N	Mean	St Dev	N	Mean	St Dev	L	Low CL	Upp CL
PcLiv	8	7.69	1.14	8	7.47	0.70	4	7.12	0.29	0.461538	-0.84	1.76
Log(ALT)	8	2.71	0.39	8	2.40	0.20	4	2.10	0.24	0.458	0.02	0.90
Log(AST)	8	2.47	0.47	8	2.41	0.15	4	2.04	0.18	0.398	-0.09	0.89
Log(SDH)	8	2.19	0.35	8	1.84	0.06	4	1.80	0.06	0.216	-0.12	0.55

[1]Relative Liver Weight calculations use untransformed data. ALT, AST and SDH calculations use Log10 transformed data.

[2]Shaded areas are statistically significant. Overall type I error rate for this study using Scheffé's method is α=0.05.

Table S3 (cont). Prop-RA Results for Binary Mixture of CHCl3 and CHBr3[1,2]

0.985:0.015 CHCl3:CHBr3
Dose = 1.0 mmol/kg/day

End Point	CHCl3			CHBr3			Mixture			L	Scheffé 95% CI	
	N	Mean	Std Dev	N	Mean	St Dev	N	Mean	St Dev		Low CL	Upp CL
PcLiv	9	5.70	0.56	9	6.42	0.63	10	6.09	0.49	-0.38271	-1.05	0.28
Log(ALT)	9	1.78	0.17	9	1.68	0.21	10	1.70	0.22	0.07462	-0.16	0.31
Log(AST)	9	1.67	0.11	9	1.73	0.10	10	1.60	0.15	0.06196	-0.08	0.21
Log(SDH)	9	1.63	0.13	9	1.57	0.15	10	1.54	0.15	0.0951	-0.08	0.27

0.985:0.015 CHCl3:CHBr3
Dose = 3.0 mmol/kg/day

End Point	CHCl3			CHBr3			Mixture			L	Scheffé 95% CI	
	N	Mean	Std Dev	N	Mean	St Dev	N	Mean	St Dev		Low CL	Upp CL
PcLiv	8	7.69	1.14	8	7.47	0.70	6	7.23	1.77	0.456933	-1.28	2.19
Log(ALT)	8	2.71	0.39	8	2.40	0.20	5	2.84	0.30	-0.12974	-0.59	0.33
Log(AST)	8	2.47	0.47	8	2.41	0.15	5	2.65	0.41	-0.1869	-0.74	0.36
Log(SDH)	8	2.19	0.35	8	1.84	0.06	5	2.17	0.42	0.01781	-0.42	0.46

[1]Relative Liver Weight calculations use untransformed data. ALT, AST and SDH calculations use Log10 transformed data.

[2]Shaded areas are statistically significant. Overall type I error rate for this study using Scheffé's method is α=0.05.

Table S4. Prop-RA Results for Binary Mixture of BDCM and CHBr3[1,2]

0.5:0.5 BDCM:CHBr3
Dose = 0.1 mmol/kg/day

	BDCM			CHBr3			Mixture				Scheffé 95% CI	
End Point	N	Mean	Std Dev	N	Mean	St Dev	N	Mean	St Dev	L	Low CL	Upp CL
PcLiv	9	4.98	0.32	11	5.23	1.29	10	5.00	0.65	0.10586	-0.79	1.00
Log(ALT)	9	1.32	0.15	11	1.48	0.27	10	1.37	0.13	0.032	-0.17	0.23
Log(AST)	9	1.53	0.07	11	1.65	0.21	10	1.67	0.25	-0.0855	-0.28	0.11
Log(SDH)	9	1.20	0.06	11	1.25	0.19	10	1.20	0.07	0.025	-0.10	0.15

0.5:0.5 BDCM:CHBr3
Dose = 1.0 mmol/kg/day

	BDCM			CHBr3			Mixture				Scheffé 95% CI	
End Point	N	Mean	Std Dev	N	Mean	St Dev	N	Mean	St Dev	L	Low CL	Upp CL
PcLiv	10	5.99	0.45	9	6.39	0.37	7	6.87	0.92	-0.68016	-1.36	0.002
Log(ALT)	10	1.96	0.29	8	1.77	0.29	6	1.87	0.45	-0.001	-0.41	0.41
Log(AST)	10	1.79	0.22	8	1.75	0.19	6	1.86	0.31	-0.088	-0.38	0.20
Log(SDH)	10	1.73	0.15	8	1.54	0.17	6	1.56	0.29	0.0725	-0.17	0.32

0.5:0.5 BDCM:CHBr3
Dose = 3.0 mmol/kg/day

	BDCM			CHBr3			Mixture				Scheffé 95% CI	
End Point	N	Mean	Std Dev	N	Mean	St Dev	N	Mean	St Dev	L	Low CL	Upp CL
PcLiv	5	8.34	1.16	4	7.69	1.13	6	7.69	0.49	0.323436	-1.05	1.69
Log(ALT)	5	2.84	0.19	4	2.44	0.35	6	2.37	0.22	0.2725	-0.10	0.64
Log(AST)	5	2.75	0.23	4	2.40	0.34	6	2.27	0.18	0.3	-0.06	0.66
Log(SDH)	5	2.53	0.33	4	2.05	0.46	6	1.79	0.09	0.5005	0.05	0.95

[1]Relative Liver Weight calculations use untransformed data. ALT, AST and SDH calculations use Log10 transformed data.

[2]Shaded areas are statistically significant. Overall type I error rate for this study using Scheffé's method is α=0.05.

Table S4 (cont). Prop-RA Results for Binary Mixture of BDCM and CHBr3[1,2]

0.96:0.040 BDCM:CHBr3
Dose = 1.0 mmol/kg/day

End Point	BDCM			CHBr3			Mixture			L	Scheffé 95% CI	
	N	Mean	Std Dev	N	Mean	St Dev	N	Mean	St Dev		Low CL	Upp CL
PcLiv	10	5.99	0.45	9	6.39	0.37	8	5.98	0.33	0.026164	-0.45	0.50
Log(ALT)	10	1.96	0.29	8	1.77	0.29	8	2.03	0.35	-0.0766	-0.45	0.30
Log(AST)	10	1.79	0.22	8	1.75	0.19	8	1.83	0.23	-0.04944	-0.31	0.21
Log(SDH)	10	1.73	0.15	8	1.54	0.17	8	1.73	0.19	-0.0124	-0.22	0.20

0.96:0.040 BDCM:CHBr3
Dose = 3.0 mmol/kg/day

End Point	BDCM			CHBr3			Mixture			L	Scheffé 95% CI	
	N	Mean	Std Dev	N	Mean	St Dev	N	Mean	St Dev		Low CL	Upp CL
PcLiv	5	8.34	1.16	4	7.69	1.13	6	8.75	0.97	-0.43673	-2.21	1.34
Log(ALT)	5	2.84	0.19	4	2.44	0.35	6	2.70	0.31	0.1248	-0.35	0.60
Log(AST)	5	2.75	0.23	4	2.40	0.34	6	2.51	0.26	0.219	-0.24	0.67
Log(SDH)	5	2.53	0.33	4	2.05	0.46	6	2.18	0.45	0.33252	-0.35	1.02

[1]Relative Liver Weight calculations use untransformed data. ALT, AST and SDH calculations use Log10 transformed data.

[2]Shaded areas are statistically significant. Overall type I error rate for this study using Scheffé's method is α=0.05.

Table S5. Prop-RA Results for Binary Mixture of BDCM and CDBM[1,2]

0.5:0.5 BDCM:CDBM
Dose = 0.1 mmol/kg/day

End Point	BDCM			CDBM			Mixture			L	Scheffé 95% CI	
	N	Mean	Std Dev	N	Mean	St Dev	N	Mean	St Dev		Low CL	Upp CL
PcLiv	7	4.69	0.63	7	5.24	0.27	7	5.05	0.42	-0.09003	-0.66	0.48
Log(ALT)	6	1.54	0.10	6	1.36	0.18	7	1.39	0.08	0.056	-0.10	0.22
Log(AST)	6	1.79	0.11	6	1.63	0.05	7	1.62	0.03	0.085	-0.01	0.18
Log(SDH)	6	1.52	0.13	6	1.42	0.08	7	1.44	0.06	0.0315	-0.09	0.15

0.5:0.5 BDCM:CDBM
Dose = 1.0 mmol/kg/day

End Point	BDCM			CDBM			Mixture			L	Scheffé 95% CI	
	N	Mean	Std Dev	N	Mean	St Dev	N	Mean	St Dev		Low CL	Upp CL
PcLiv	6	5.94	0.44	7	6.09	0.48	2	6.48	0.24	-0.46903	-1.42	0.48
Log(ALT)	6	1.90	0.44	7	1.93	0.44	2	2.00	0.34	-0.085	-1.00	0.83
Log(AST)	6	1.92	0.29	7	1.93	0.23	2	1.90	0.31	0.023	-0.54	0.58
Log(SDH)	6	1.95	0.35	7	1.88	0.33	2	1.89	0.01	0.024	-0.66	0.71

0.5:0.5 BDCM:CDBM
Dose = 3.0 mmol/kg/day

End Point	BDCM			CDBM			Mixture			L	Scheffé 95% CI	
	N	Mean	Std Dev	N	Mean	St Dev	N	Mean	St Dev		Low CL	Upp CL
PcLiv	5	8.78	0.59	2	5.86	0.51	3	8.05	1.11	-0.72649	-2.41	0.96
Log(ALT)	5	3.13	0.08	2	2.90	0.01	3	2.64	0.38	0.37	-0.09	0.83
Log(AST)	5	2.96	0.12	2	3.03	0.06	3	2.61	0.28	0.3885	-0.002	0.78
Log(SDH)	5	2.67	0.19	2	2.53	0.05	3	2.31	0.32	0.2885	-0.21	0.78

[1]Relative Liver Weight calculations use untransformed data. ALT, AST and SDH calculations use Log10 transformed data.

[2]Shaded areas are statistically significant. Overall type I error rate for this study using Scheffé's method is α=0.05.

Table S5 (cont). Prop-RA Results Binary Mixture of BDCM and CDBM[1,2]

0.706:0.294 BDCM:CDBM
Dose = 1.0 mmol/kg/day

	BDCM			CDBM			Mixture				Scheffé 95% CI	
End Point	N	Mean	Std Dev	N	Mean	St Dev	N	Mean	St Dev	L	Low CL	Upp CL
PcLiv	6	5.94	0.44	7	6.09	0.48	7	5.78	0.30	0.205249	-0.34	0.75
Log(ALT)	6	1.90	0.44	7	1.93	0.44	7	2.02	0.44	-0.10959	-0.68	0.46
Log(AST)	6	1.92	0.29	7	1.93	0.23	7	1.93	0.35	-0.00665	-0.39	0.38
Log(SDH)	6	1.95	0.35	7	1.88	0.33	7	1.94	0.31	-0.00693	-0.44	0.43

0.706:0.294 BDCM:CDBM
Dose = 3.0 mmol/kg/day

	BDCM			CDBM			Mixture				Scheffé 95% CI	
End Point	N	Mean	Std Dev	N	Mean	St Dev	N	Mean	St Dev	L	Low CL	Upp CL
PcLiv	5	8.78	0.59	2	5.86	0.51	3	10.19	1.33	-2.27129	-4.11	-0.44
Log(ALT)	5	3.13	0.08	2	2.90	0.01	3	3.11	0.44	-0.05121	-0.57	0.47
Log(AST)	5	2.96	0.12	2	3.03	0.06	3	2.96	0.49	0.021286	-0.57	0.61
Log(SDH)	5	2.67	0.19	2	2.53	0.05	3	2.68	0.15	-0.04645	-0.40	0.31

[1]PcLiv calculations use untransformed data. ALT, AST and SDH calculations use Log10 transformed data.

[2]Shaded areas are statistically significant. Overall type I error rate for this study using Scheffé's method is α=0.05.

Table S6. Prop-RA Results for Binary Mixture of BDCM and CDBM-rep[1,2]

0.5:0.5 BDCM:CDBM
Dose = 0.1 mmol/kg/day

End Point	BDCM			CDBM			Mixture			L	Scheffé 95% CI	
	N	Mean	Std Dev	N	Mean	St Dev	N	Mean	St Dev		Low CL	Upp CL
PcLiv	8	5.27	0.48	11	5.01	0.44	7	5.07	0.48	0.072058	-0.47	0.61
Log(ALT)	8	1.43	0.10	11	1.28	0.14	7	1.36	0.14	-0.004	-0.15	0.14
Log(AST)	8	1.62	0.07	11	1.59	0.11	7	1.61	0.10	-0.008	-0.12	0.10
Log(SDH)	8	1.39	0.06	11	1.35	0.06	7	1.36	0.07	0.0065	-0.06	0.08

0.5:0.5 BDCM:CDBM
Dose = 1.0 mmol/kg/day

End Point	BDCM			CDBM			Mixture			L	Scheffé 95% CI	
	N	Mean	Std Dev	N	Mean	St Dev	N	Mean	St Dev		Low CL	Upp CL
PcLiv	7	6.14	0.37	8	6.19	0.42	5	6.57	0.67	-0.40968	-1.06	0.24
Log(ALT)	7	1.89	0.26	8	1.82	0.22	5	1.91	0.33	-0.059	-0.42	0.31
Log(AST)	7	1.82	0.27	8	1.83	0.16	5	1.83	0.26	-0.004	-0.32	0.31
Log(SDH)	7	1.83	0.15	8	1.80	0.18	5	1.91	0.33	-0.097	-0.39	0.20

0.5:0.5 BDCM:CDBM
Dose = 3.0 mmol/kg/day

End Point	BDCM			CDBM			Mixture			L	Scheffé 95% CI	
	N	Mean	Std Dev	N	Mean	St Dev	N	Mean	St Dev		Low CL	Upp CL
PcLiv	4	8.81	0.38	1	8.74	.	1	7.81	.	0.964414	N/A	N/A
Log(ALT)	4	2.00	0.78	1	2.82	.	1	2.17	.	0.2435	N/A	N/A
Log(AST)	4	2.32	0.29	1	2.72	.	1	2.11	.	0.407	N/A	N/A
Log(SDH)	4	2.58	0.03	1	2.57	.	1	2.02	.	0.5595	N/A	N/A

[1]Relative Liver Weight calculations use untransformed data. ALT, AST and SDH calculations use Log10 transformed data.
[2]Shaded areas are statistically significant. Overall type I error rate for this study using Scheffé's method is α=0.05.

Table S6 (cont). Prop-RA Results for Binary Mixture of BDCM and CDBM-rep[1,2]

0.706:0.294 BDCM:CDBM
Dose = 1.0 mmol/kg/day

	BDCM			CDBM			Mixture				Scheffé 95% CI	
End Point	N	Mean	Std Dev	N	Mean	St Dev	N	Mean	St Dev	L	Low CL	Upp CL
PcLiv	7	6.14	0.37	8	6.19	0.42	8	6.17	0.35	-0.01415	-0.47	0.44
Log(ALT)	7	1.89	0.26	8	1.82	0.22	8	1.76	0.11	0.109244	-0.13	0.35
Log(AST)	7	1.82	0.27	8	1.83	0.16	8	1.74	0.06	0.081176	-0.13	0.30
Log(SDH)	7	1.83	0.15	8	1.80	0.18	8	1.80	0.10	0.022592	-0.15	0.19

0.706:0.294 BDCM:CDBM
Dose = 3.0 mmol/kg/day

	BDCM			CDBM			Mixture				Scheffé 95% CI	
End Point	N	Mean	Std Dev	N	Mean	St Dev	N	Mean	St Dev	L	Low CL	Upp CL
PcLiv	4	8.81	0.38	1	8.74	.	6	8.02	0.64	0.771884	N/A	N/A
Log(ALT)	4	2.00	0.78	1	2.82	.	6	2.51	0.18	-0.26239	N/A	N/A
Log(AST)	4	2.32	0.29	1	2.72	.	6	2.38	0.16	0.051776	N/A	N/A
Log(SDH)	4	2.58	0.03	1	2.57	.	6	2.28	0.31	0.303178	N/A	N/A

[1]Relative Liver Weight calculations use untransformed data. ALT, AST and SDH calculations use Log10 transformed data.

[2]Shaded areas are statistically significant. Overall type I error rate for this study using Scheffé's method is α=0.05.

Table S7. Prop-RA Results for Binary Mixture of CHCl3 and CDBM[1,2]

0.5:0.5 CHCl3:CDBM
Dose = 0.1 mmol/kg/day

	CHCl3			CDBM			Mixture				Scheffé 95% CI	
End Point	N	Mean	Std Dev	N	Mean	St Dev	N	Mean	St Dev	L	Low CL	Upp CL
PcLiv	8	4.50	0.27	8	4.88	0.52	8	4.76	0.89	-0.06814	-0.77	0.63
Log(ALT)	8	1.49	0.22	8	1.41	0.07	7	1.47	0.10	-0.019	-0.20	0.16
Log(AST)	8	1.57	0.05	8	1.56	0.09	7	1.69	0.06	-0.126	-0.21	-0.04
Log(SDH)	8	1.26	0.07	8	1.25	0.06	7	1.37	0.15	-0.114	-0.23	0.01

0.5:0.5 CHCl3:CDBM
Dose = 1.0 mmol/kg/day

	CHCl3			CDBM			Mixture				Scheffé 95% CI	
End Point	N	Mean	Std Dev	N	Mean	St Dev	N	Mean	St Dev	L	Low CL	Upp CL
PcLiv	8	6.09	0.29	10	6.31	0.43	9	6.01	0.58	0.189974	-0.29	0.67
Log(ALT)	8	1.84	0.29	10	1.74	0.26	9	1.83	0.19	-0.045	-0.31	0.22
Log(AST)	8	1.77	0.22	10	1.75	0.24	9	1.86	0.27	-0.105	-0.37	0.16
Log(SDH)	8	1.61	0.28	10	1.68	0.16	8	1.73	0.19	-0.085	-0.32	0.15

0.5:0.5 CHCl3:CDBM
Dose = 3.0 mmol/kg/day

	CHCl3			CDBM			Mixture				Scheffé 95% CI	
End Point	N	Mean	Std Dev	N	Mean	St Dev	N	Mean	St Dev	L	Low CL	Upp CL
PcLiv	4	6.74	1.26	3	8.05	0.76	5	7.77	1.00	-0.37062	-2.17	1.43
Log(ALT)	4	1.68	0.73	3	2.25	0.18	5	2.29	0.81	-0.33	-1.51	0.85
Log(AST)	4	2.29	0.49	3	2.58	0.15	5	2.70	0.36	-0.267	-0.91	0.38
Log(SDH)	4	2.27	0.64	3	2.37	0.29	5	2.50	0.34	-0.1845	-0.96	0.59

[1]Relative Liver Weight calculations use untransformed data. ALT, AST and SDH calculations use Log10 transformed data.
[2]Shaded areas are statistically significant. Overall type I error rate for this study using Scheffé's method is α=0.05.

Table S7 (cont). Prop-RA Results for Binary Mixture of CHCl3 and CDBM[1,2]

0.867:0.133 CHCl3:CDBM
Dose = 1.0 mmol/kg/day

End Point	CHCl3			CDBM			Mixture			L	Scheffé 95% CI	
	N	Mean	Std Dev	N	Mean	St Dev	N	Mean	St Dev		Low CL	Upp CL
PcLiv	8	6.09	0.29	10	6.31	0.43	8	6.01	0.42	0.110483	-0.37	0.59
Log(ALT)	8	1.84	0.29	10	1.74	0.26	8	1.77	0.39	0.0517	-0.34	0.44
Log(AST)	8	1.77	0.22	10	1.75	0.24	8	1.73	0.25	0.034074	-0.26	0.32
Log(SDH)	8	1.61	0.28	10	1.68	0.16	8	1.61	0.25	0.010044	-0.27	0.29

0.867:0.133 CHCl3:CDBM
Dose = 3.0 mmol/kg/day

End Point	CHCl3			CDBM			Mixture			L	Scheffé 95% CI	
	N	Mean	Std Dev	N	Mean	St Dev	N	Mean	St Dev		Low CL	Upp CL
PcLiv	4	6.74	1.26	3	8.05	0.76	5	7.71	0.51	-0.78663	-2.40	0.82
Log(ALT)	4	1.68	0.73	3	2.25	0.18	5	2.19	0.13	-0.42899	-1.23	0.37
Log(AST)	4	2.29	0.49	3	2.58	0.15	5	2.27	0.26	0.061102	-0.56	0.68
Log(SDH)	4	2.27	0.64	3	2.37	0.29	5	2.32	0.32	-0.0443	-0.86	0.77

[1]Relative Liver Weight calculations use untransformed data. ALT, AST and SDH calculations use Log10 transformed data.
[2]Shaded areas are statistically significant. Overall type I error rate for this study using Scheffé's method is $\alpha=0.05$.

Table S8. Prop-RA Results for Binary Mixture of CDBM and CHBr3[1,2]

0.5:0.5 CDBM and CHBr3
Dose = 0.1 mmol/kg/day

	CDBM			CHBr3			Mixture				Scheffé 95% CI	
End Point	N	Mean	Std Dev	N	Mean	St Dev	N	Mean	St Dev	L	Low CL	Upp CL
PcLiv	11	5.02	0.39	11	5.08	0.25	8	5.27	0.36	-0.21684	-0.58	0.14
Log(ALT)	11	1.29	0.04	11	1.33	0.12	9	1.29	0.08	0.027	-0.06	0.12
Log(AST)	11	1.64	0.14	11	1.61	0.18	9	1.55	0.07	0.078	-0.07	0.23
Log(SDH)	11	1.14	0.05	11	1.14	0.07	9	1.14	0.06	0	-0.06	0.06

0.5:0.5 CDBM and CHBr3
Dose = 1.0 mmol/kg/day

	CDBM			CHBr3			Mixture				Scheffé 95% CI	
End Point	N	Mean	Std Dev	N	Mean	St Dev	N	Mean	St Dev	L	Low CL	Upp CL
PcLiv	10	6.40	0.24	11	6.43	0.33	7	6.58	0.46	-0.16748	-0.55	0.22
Log(ALT)	10	1.70	0.26	11	1.57	0.20	7	1.54	0.12	0.093	-0.15	0.33
Log(AST)	10	1.76	0.21	11	1.68	0.13	7	1.68	0.06	0.038	-0.14	0.22
Log(SDH)	10	1.44	0.16	11	1.35	0.17	7	1.33	0.08	0.067	-0.10	0.23

0.5:0.5 CDBM and CHBr3
Dose = 3.0 mmol/kg/day

	CDBM			CHBr3			Mixture				Scheffé 95% CI	
End Point	N	Mean	Std Dev	N	Mean	St Dev	N	Mean	St Dev	L	Low CL	Upp CL
PcLiv	5	7.59	0.46	4	7.55	0.58	5	6.96	0.52	0.611068	-0.20	1.42
Log(ALT)	5	2.82	0.20	4	2.19	0.23	5	2.23	0.35	0.2755	-0.15	0.70
Log(AST)	5	2.74	0.22	4	2.30	0.19	5	2.28	0.25	0.239	-0.11	0.59
Log(SDH)	5	2.08	0.28	4	1.76	0.17	5	1.85	0.34	0.0675	-0.38	0.51

[1]Relative Liver Weight calculations use untransformed data. ALT, AST and SDH calculations use Log10 transformed data.

[2]Shaded areas are statistically significant. Overall type I error rate for this study using Scheffé's method is α=0.05.

Table S8 (cont). Prop-RA Results for Binary Mixture of CDBM and CHBr3[1,2]

0.909:0.091 CDBM:CHBr3
Dose = 1.0 mmol/kg/day

End Point	CDBM			CHBr3			Mixture			L	Scheffé 95% CI	
	N	Mean	Std Dev	N	Mean	St Dev	N	Mean	St Dev		Low CL	Upp CL
PcLiv	10	6.40	0.24	11	6.43	0.33	9	6.30	0.41	0.100385	-0.28	0.48
Log(ALT)	10	1.70	0.26	11	1.57	0.20	9	1.64	0.25	0.04917	-0.22	0.32
Log(AST)	10	1.76	0.21	11	1.68	0.13	9	1.74	0.20	0.014356	-0.20	0.22
Log(SDH)	10	1.44	0.16	11	1.35	0.17	9	1.38	0.19	0.049174	-0.15	0.24

0.909:0.091 CDBM:CHBr3
Dose = 3.0 mmol/kg/day

End Point	CDBM			CHBr3			Mixture			L	Scheffé 95% CI	
	N	Mean	Std Dev	N	Mean	St Dev	N	Mean	St Dev		Low CL	Upp CL
PcLiv	5	7.59	0.46	4	7.55	0.58	2	8.52	0.36	-0.93904	-2.15	0.27
Log(ALT)	5	2.82	0.20	4	2.19	0.23	2	2.19	0.10	0.570307	0.08	1.06
Log(AST)	5	2.74	0.22	4	2.30	0.19	2	2.11	0.17	0.590324	0.10	1.08
Log(SDH)	5	2.08	0.28	4	1.76	0.17	2	1.77	0.16	0.277335	-0.29	0.85

[1]Relative Liver Weight calculations use untransformed data. ALT, AST and SDH calculations use Log10 transformed data.

[2]Shaded areas are statistically significant. Overall type I error rate for this study using Scheffé's method is α=0.05.

Table S9. Prop-RA Untransformed Data Results for Binary Mixture of CHCl3 and BDCM[1,2]

0.5:0.5 CHCl3:BDCM
Dose = 0.1 mmol/kg/day

End Point	Chem A			Chem B			Mixture			L	Scheffe 95% Confidence	
	N	Mean	Std Dev	N	Mean	St Dev	N	Mean	St Dev		Lower CL	Upper CL
Rel Liver Wt	10	4.66	0.48	10	4.72	0.40	11	4.98	0.49	-0.28717	-0.74	0.16
ALT	10	26.30	5.76	10	26.10	7.56	11	25.82	6.08	0.381818	-5.92	6.69
AST	10	41.20	8.59	10	45.80	12.61	11	43.36	10.64	0.136364	-10.28	10.55
SDH	10	14.41	1.16	10	12.66	3.45	11	14.50	1.90	-0.965	-3.25	1.32

0.5:0.5 CHCl3:BDCM
Dose = 1.0 mmol/kg/day

End Point	Chem A			Chem B			Mixture			L	Scheffe 95% Confidence	
	N	Mean	Std Dev	N	Mean	St Dev	N	Mean	St Dev		Lower CL	Upper CL
Rel Liver Wt	9	5.40	0.31	7	6.15	0.83	10	5.76	0.37	0.018606	-0.53	0.56
ALT	9	43.44	14.56	7	94.86	60.82	11	45.00	14.01	24.15079	-9.49	57.79
AST	9	44.00	8.09	7	64.43	25.86	11	42.82	7.17	11.3961	-3.47	26.26
SDH	9	21.43	3.57	7	34.14	20.69	11	24.06	6.04	3.724459	-7.81	15.25

0.5:0.5 CHCl3:BDCM
Dose = 3.0 mmol/kg/day

End Point	Chem A			Chem B			Mixture			L	Scheffe 95% Confidence	
	N	Mean	Std Dev	N	Mean	St Dev	N	Mean	St Dev		Lower CL	Upper CL
Rel Liver Wt	7	7.72	0.95	7	8.16	1.59	9	8.42	0.93	-0.47626	-1.80	0.85
ALT	7	630.86	461.50	7	989.00	749.65	9	948.44	737.60	-138.516	-896.12	619.08
AST	7	418.43	436.43	7	941.14	810.13	9	847.22	885.45	-167.437	-1018.22	683.35
SDH	7	104.73	68.50	7	133.59	158.15	9	155.68	144.59	-36.5206	-184.93	111.89

[1]All calculations use original untransformed data.

[2]Shaded areas are statistically significant. Overall type I error rate for this study using Scheffé's method is α=0.05.

Table S9 (cont). Prop-RA Untransformed Data Results for Binary Mixture of CHCl3 and BDCM[1,2]

0.730:0.270 CHCl3:BDCM
Dose = 1.0 mmol/kg/day

End Point	Chem A			Chem B			Mixture			L	Scheffe 95% Confidence	
	N	Mean	Std Dev	N	Mean	St Dev	N	Mean	St Dev		Lower CL	Upper CL
Rel Liver Wt	9	5.40	0.31	7	6.15	0.83	11	5.68	0.44	-0.07441	-0.63	0.48
ALT	9	43.44	14.56	7	94.86	60.82	11	64.73	23.72	-7.4014	-44.06	29.25
AST	9	44.00	8.09	7	64.43	25.86	11	64.82	9.87	-15.3025	-31.14	0.53
SDH	9	21.43	3.57	7	34.14	20.69	11	26.38	8.75	-1.51691	-14.03	10.99

0.730:0.270 CHCl3:BDCM
Dose = 3.0 mmol/kg/day

End Point	Chem A			Chem B			Mixture			L	Scheffe 95% Confidence	
	N	Mean	Std Dev	N	Mean	St Dev	N	Mean	St Dev		Lower CL	Upper CL
Rel Liver Wt	7	7.72	0.95	7	8.16	1.59	11	8.11	0.85	-0.26488	-1.51	0.98
ALT	7	630.86	461.50	7	989.00	749.65	11	1128.55	869.60	-400.994	-1224.61	422.62
AST	7	418.43	436.43	7	941.14	810.13	11	718.27	536.39	-158.711	-823.57	506.15
SDH	7	104.73	68.50	7	133.59	158.15	11	141.90	101.32	-29.38	-154.29	95.53

[1]All calculations use original untransformed data.

[2]Shaded areas are statistically significant. Overall type I error rate for this study using Scheffé's method is α=0.05.

Table S10. Prop-RA Untransformed Data Results for Binary Mixture of CHCl3 and BDCM-rep[1,2]

0.5:0.5 CHCl3:BDCM
Dose = 0.1 mmol/kg/day

End Point	CHCl3			BDCM			Mixture			L	Confidence Interval	
	N	Mean	Std Dev	N	Mean	St Dev	N	Mean	St Dev		Lower CL	Upper CL
Wt	8	5.18	0.39	10	5.38	0.39	10	5.23	0.37	0.053491	-0.34	0.45
ALT	8	25.63	9.43	10	23.30	8.37	10	25.30	7.12	-0.8375	-9.34	7.66
AST	8	40.38	7.84	10	41.50	10.64	10	38.70	7.06	2.2375	-6.72	11.20
SDH	8	13.83	3.12	10	12.52	2.18	10	13.35	2.08	-0.1775	-2.69	2.34

0.5:0.5 CHCl3:BDCM
Dose = 1.0 mmol/kg/day

End Point	CHCl3			BDCM			Mixture			L	Confidence Interval	
	N	Mean	Std Dev	N	Mean	St Dev	N	Mean	St Dev		Lower CL	Upper CL
Wt	9	6.34	0.54	8	6.22	0.50	10	6.30	0.63	-0.01859	-0.61	0.57
ALT	9	52.56	16.44	7	77.57	39.09	9	61.22	35.15	3.84127	-30.21	37.89
AST	9	45.44	5.59	7	59.29	17.92	9	56.67	18.94	-4.30159	-20.91	12.31
SDH	9	28.04	10.52	7	43.97	18.10	9	32.69	14.97	3.319048	-12.61	19.25

0.5:0.5 CHCl3:BDCM
Dose = 3.0 mmol/kg/day

End Point	CHCl3			BDCM			Mixture			L	Confidence Interval	
	N	Mean	Std Dev	N	Mean	St Dev	N	Mean	St Dev		Lower CL	Upper CL
Wt	5	8.29	1.78	7	8.41	0.55	9	9.22	1.77	-0.86813	-2.62	0.88
ALT	3	1205.00	1057.60	7	641.86	264.05	10	1508.30	1258.59	-584.871	-1835.98	666.24
AST	3	633.33	596.96	7	478.71	215.89	10	1141.00	1324.22	-584.976	-1830.99	661.04
SDH	3	301.27	253.79	7	188.46	82.55	10	333.01	341.88	-88.1481	-424.40	248.11

[1]All calculations use original untransformed data.

[2]Shaded areas are statistically significant. Overall type I error rate for this study using Scheffé's method is α=0.05.

Table S10 (cont). Prop-RA Untransformed Data Results for Binary Mixture of CHCl3 and BDCM-rep[1,2]

0.730:0.270 CHCl3:BDCM

Dose = 1.0 mmol/kg/day

End Point	CHCl3			BDCM			Mixture			L	Confidence Interval	
	N	Mean	Std Dev	N	Mean	St Dev	N	Mean	St Dev		Lower CL	Upper CL
Wt	9	6.34	0.54	8	6.22	0.50	10	6.40	0.72	-0.0947	-0.74	0.55
ALT	9	52.56	16.44	7	77.57	39.09	10	44.20	14.73	15.10984	-10.78	41.00
AST	9	45.44	5.59	7	59.29	17.92	10	46.00	10.28	3.181587	-9.39	15.75
SDH	9	28.04	10.52	7	43.97	18.10	10	23.86	7.60	8.48473	-4.56	21.53

0.730:0.270 CHCl3:BDCM

Dose = 3.0 mmol/kg/day

End Point	CHCl3			BDCM			Mixture			L	Confidence Interval	
	N	Mean	Std Dev	N	Mean	St Dev	N	Mean	St Dev		Lower CL	Upper CL
Wt	5	8.29	1.78	7	8.41	0.55	7	8.43	0.98	-0.11181	-1.66	1.43
ALT	3	1205.00	1057.60	7	641.86	264.05	7	855.57	769.66	197.38	-850.25	1245.01
AST	3	633.33	596.96	7	478.71	215.89	7	657.00	627.80	-65.4138	-835.72	704.89
SDH	3	301.27	253.79	7	188.46	82.55	7	240.03	216.22	30.77952	-251.30	312.86

[1]All calculations use original untransformed data.

[2]Shaded areas are statistically significant. Overall type I error rate for this study using Scheffé's method is α=0.05.

Table S11. Prop-RA Untransformed Data Results for Binary Mixture of CHCl3 and CHBr3[1,2]

0.5:0.5 CHCl3:CHBr3
Dose = 0.1 mmol/kg/day

End Point	Chem A			Chem B			Mixture			L	Confidence Interval	
	N	Mean	Std Dev	N	Mean	St Dev	N	Mean	St Dev		CL	Upper CL
Rel Liver Wt	10	4.97	0.62	4	5.06	0.33	11	4.75	0.40	0.268194	-0.28	0.81
ALT	10	27.10	10.72	17	29.44	5.77	10	28.90	9.55	-0.62778	-8.65	7.39
AST	10	43.50	12.29	34	45.33	13.59	10	42.50	7.93	1.916667	-9.59	13.42
SDH	9	19.34	3.28	12	19.94	3.33	10	20.91	3.74	-1.26556	-4.70	2.17

0.5:0.5 CHCl3:CHBr3
Dose = 1.0 mmol/kg/day

End Point	Chem A			Chem B			Mixture			L	Confidence Interval	
	N	Mean	Std Dev	N	Mean	St Dev	N	Mean	St Dev		CL	Upper CL
Rel Liver Wt	9	5.70	0.56	9	6.42	0.63	9	6.08	0.39	-0.01351	-0.58	0.56
ALT	9	64.11	27.72	9	54.00	28.57	9	52.89	26.49	6.166667	-23.24	35.57
AST	9	47.44	11.92	9	55.00	13.96	9	51.78	15.43	-0.55556	-15.30	14.19
SDH	9	44.77	13.52	9	39.71	14.71	9	34.32	5.94	7.916667	-4.90	20.73

0.5:0.5 CHCl3:CHBr3
Dose = 3.0 mmol/kg/day

End Point	Chem A			Chem B			Mixture			L	Confidence Interval	
	N	Mean	Std Dev	N	Mean	St Dev	N	Mean	St Dev		CL	Upper CL
Rel Liver Wt	8	7.69	1.14	8	7.47	0.70	4	7.12	0.29	0.461538	-0.84	1.76
ALT	8	728.13	616.68	8	272.75	118.97	4	138.75	63.84	361.6875	-243.45	966.83
AST	8	490.50	499.45	8	268.75	88.78	4	116.50	43.88	263.125	-225.36	751.61
SDH	8	208.94	187.35	8	70.13	8.55	4	63.53	9.09	76.00625	-104.39	256.40

[1]All calculations use original untransformed data.

[2]Shaded areas are statistically significant. Overall type I error rate for this study using Scheffé's method is α=0.05.

Table S11 (cont). Prop-RA Untransformed Data Results for Binary Mixture of CHCl3 and CHBr3[1,2]

0.985:0.015 CHCl3:CHBr3
Dose = 1.0 mmol/kg/day

End Point	Chem A			Chem B			Mixture			L	Confidence Interval	
	N	Mean	Std Dev	N	Mean	St Dev	N	Mean	St Dev		CL	Upper CL
Rel Liver Wt	9	5.70	0.56	9	6.42	0.63	10	6.09	0.49	-0.38271	-1.05	0.28
ALT	9	64.11	27.72	9	54.00	28.57	10	56.40	30.96	7.559444	-27.07	42.19
AST	9	47.44	11.92	9	55.00	13.96	10	42.40	14.95	5.157778	-11.12	21.44
SDH	9	44.77	13.52	9	39.71	14.71	10	36.45	12.71	8.240833	-7.93	24.41

0.985:0.015 CHCl3:CHBr3
Dose = 3.0 mmol/kg/day

End Point	Chem A			Chem B			Mixture			L	Confidence Interval	
	N	Mean	Std Dev	N	Mean	St Dev	N	Mean	St Dev		CL	Upper CL
Rel Liver Wt	8	7.69	1.14	8	7.47	0.70	6	7.23	1.77	0.456933	-1.28	2.19
ALT	8	728.13	616.68	8	272.75	118.97	5	836.00	588.73	-114.706	-840.19	610.78
AST	8	490.50	499.45	8	268.75	88.78	5	689.60	821.54	-202.426	-958.19	553.34
SDH	8	208.94	187.35	8	70.13	8.55	5	210.74	188.71	-3.88469	-225.96	218.20

[1]All calculations use original untransformed data.

[2]Shaded areas are statistically significant. Overall type I error rate for this study using Scheffé's method is α=0.05.

Table S12. Prop-RA Untransformed Data Results for Binary Mixture of BDCM and CHBr3[1,2]

0.5:0.5 BDCM:CHBr3
Dose = 0.1 mmol/kg/day

End Point	Chem A			Chem B			Mixture			L	Confidence Interval	
	N	Mean	Std Dev	N	Mean	St Dev	N	Mean	St Dev		CL	Upper CL
Rel Liver Wt	9	4.98	0.32	11	5.23	1.29	10	5.00	0.65	0.10586	-0.79	1.00
ALT	9	22.22	7.74	11	39.27	45.02	10	24.30	7.07	6.447475	-21.71	34.60
AST	9	34.00	5.50	11	51.45	37.21	10	55.80	39.80	-13.0727	-45.63	19.48
SDH	9	16.06	2.34	11	20.20	14.38	10	16.11	2.70	2.017778	-7.01	11.04

0.5:0.5 BDCM:CHBr3
Dose = 1.0 mmol/kg/day

End Point	Chem A			Chem B			Mixture			L	Confidence Interval	
	N	Mean	Std Dev	N	Mean	St Dev	N	Mean	St Dev		CL	Upper CL
Rel Liver Wt	10	5.99	0.45	9	6.39	0.37	7	6.87	0.92	-0.68016	-1.36	0.00
ALT	10	110.80	72.03	8	73.13	56.93	6	111.17	99.36	-19.2042	-112.69	74.28
AST	10	69.00	40.90	8	61.88	34.08	6	86.67	53.57	-21.2292	-73.79	31.33
SDH	10	56.28	18.33	8	37.48	15.38	6	43.07	24.74	3.810833	-20.06	27.68

0.5:0.5 BDCM:CHBr3
Dose = 3.0 mmol/kg/day

End Point	Chem A			Chem B			Mixture			L	Confidence Interval	
	N	Mean	Std Dev	N	Mean	St Dev	N	Mean	St Dev		CL	Upper CL
Rel Liver Wt	5	8.34	1.16	4	7.69	1.13	6	7.69	0.49	0.323436	-1.05	1.69
ALT	5	754.80	333.44	4	346.50	251.46	6	259.83	132.67	290.8167	-70.57	652.20
AST	5	622.00	303.18	4	309.75	212.74	6	199.83	76.30	266.0417	-44.23	576.32
SDH	5	404.30	193.86	4	183.28	233.98	6	62.72	13.00	231.0708	-7.71	469.85

[1]All calculations use original untransformed data.

[2]Shaded areas are statistically significant. Overall type I error rate for this study using Scheffé's method is α=0.05.

Table S12 (cont). Prop-RA Untransformed Data Results for Binary Mixture of BDCM and CHBr3[1,2]

0.96:0.040 BDCM:CHBr3
Dose = 1.0 mmol/kg/day

	Chem A			Chem B			Mixture				Confidence Interval	
End Point	N	Mean	Std Dev	N	Mean	St Dev	N	Mean	St Dev	L	CL	Upper CL
Rel Liver Wt	10	5.99	0.45	9	6.39	0.37	8	5.98	0.33	0.026164	-0.45	0.50
ALT	10	110.80	72.03	8	73.13	56.93	8	141.13	107.57	-31.832	-130.46	66.79
AST	10	69.00	40.90	8	61.88	34.08	8	77.63	43.16	-8.91	-57.31	39.49
SDH	10	56.28	18.33	8	37.48	15.38	8	58.89	26.09	-3.3597	-28.08	21.36

0.96:0.040 BDCM:CHBr3
Dose = 3.0 mmol/kg/day

	Chem A			Chem B			Mixture				Confidence Interval	
End Point	N	Mean	Std Dev	N	Mean	St Dev	N	Mean	St Dev	L	CL	Upper CL
Rel Liver Wt	5	8.34	1.16	4	7.69	1.13	6	8.75	0.97	-0.43673	-2.21	1.34
ALT	5	754.80	333.44	4	346.50	251.46	6	651.83	605.20	86.63467	-662.41	835.68
AST	5	622.00	303.18	4	309.75	212.74	6	381.33	241.75	228.1767	-197.31	653.67
SDH	5	404.30	193.86	4	183.28	233.98	6	241.70	254.94	153.759	-227.71	535.23

[1]All calculations use original untransformed data.

[2]Shaded areas are statistically significant. Overall type I error rate for this study using Scheffé's method is α=0.05.

Table S13. Prop-RA Untransformed Data Results for Binary Mixture of BDCM and CDBM[1,2]

0.5:0.5 BDCM:CDBM
Dose = 0.1 mmol/kg/day

	Chem A			Chem B			Mixture				Confidence Interval	
End Point	N	Mean	Std Dev	N	Mean	St Dev	N	Mean	St Dev	L	CL	Upper CL
Rel Liver Wt	7	4.69	0.63	7	5.24	0.27	7	5.05	0.42	-0.09003	-0.66	0.48
ALT	6	35.17	7.11	6	24.33	8.52	7	25.00	4.43	4.75	-3.93	13.43
AST	6	62.67	16.97	6	42.83	5.08	7	42.00	3.06	10.75	-2.17	23.67
SDH	6	34.15	10.87	6	26.92	5.20	7	27.77	4.39	2.761905	-6.54	12.06

0.5:0.5 BDCM:CDBM
Dose = 1.0 mmol/kg/day

	Chem A			Chem B			Mixture				Confidence Interval	
End Point	N	Mean	Std Dev	N	Mean	St Dev	N	Mean	St Dev	L	CL	Upper CL
Rel Liver Wt	6	5.94	0.44	7	6.09	0.48	2	6.48	0.24	-0.46903	-1.42	0.48
ALT	6	125.83	148.05	7	130.71	119.52	2	116.00	82.02	12.27381	-262.56	287.10
AST	6	101.50	71.89	7	95.29	46.42	2	90.50	60.10	7.892857	-117.98	133.77
SDH	6	125.23	134.38	7	102.61	109.75	2	77.80	0.99	36.12381	-210.41	282.66

0.5:0.5 BDCM:CDBM
Dose = 3.0 mmol/kg/day

	Chem A			Chem B			Mixture				Confidence Interval	
End Point	N	Mean	Std Dev	N	Mean	St Dev	N	Mean	St Dev	L	CL	Upper CL
Rel Liver Wt	5	8.78	0.59	2	5.86	0.51	3	8.05	1.11	-0.72649	-2.41	0.96
ALT	5	1359.00	237.47	2	786.00	16.97	3	572.33	524.68	500.1667	-230.75	1231.08
AST	5	950.40	264.84	2	1084.50	142.13	3	469.00	306.26	548.45	-31.21	1128.11
SDH	5	509.58	231.20	2	340.65	39.67	3	239.60	135.00	185.515	-230.74	601.77

[1]All calculations use original untransformed data.

[2]Shaded areas are statistically significant. Overall type I error rate for this study using Scheffé's method is $\alpha=0.05$.

Table S13 (cont). Prop-RA Untransformed Data Results for Binary Mixture of BDCM and CDBM[1,2]

0.706:0.294 BDCM:CDBM
Dose = 1.0 mmol/kg/day

	Chem A			Chem B			Mixture				Confidence Interval	
End Point	N	Mean	Std Dev	N	Mean	St Dev	N	Mean	St Dev	L	CL	Upper CL
Rel Liver Wt	6	5.94	0.44	7	6.09	0.48	7	5.78	0.30	0.205249	-0.34	0.75
ALT	6	125.83	148.05	7	130.71	119.52	7	158.86	160.51	-31.5888	-219.27	156.09
AST	6	101.50	71.89	7	95.29	46.42	7	114.57	105.38	-14.8984	-117.91	88.11
SDH	6	125.23	134.38	7	102.61	109.75	7	109.47	88.45	9.111905	-136.12	154.35

0.706:0.294 BDCM:CDBM
Dose = 3.0 mmol/kg/day

	Chem A			Chem B			Mixture				Confidence Interval	
End Point	N	Mean	Std Dev	N	Mean	St Dev	N	Mean	St Dev	L	CL	Upper CL
Rel Liver Wt	5	8.78	0.59	2	5.86	0.51	3	10.19	1.33	-2.27129	-4.11	-0.44
ALT	5	1359.00	237.47	2	786.00	16.97	3	1714.33	1310.64	-523.792	-2060.08	1012.50
AST	5	950.40	264.84	2	1084.50	142.13	3	1288.33	1047.27	-298.505	-1566.52	969.51
SDH	5	509.58	231.20	2	340.65	39.67	3	494.13	177.96	-34.2188	-458.09	389.65

[1]All calculations use original untransformed data.

[2]Shaded areas are statistically significant. Overall type I error rate for this study using Scheffé's method is α=0.05.

Table S14. Prop-RA Untransformed Data Results for Binary Mixture of BDCM and CDBM-rep[1,2]

0.5:0.5 BDCM:CDBM
Dose = 0.1 mmol/kg/day

End Point	Chem A			Chem B			Mixture			L	Confidence Interval	
	N	Mean	Std Dev	N	Mean	St Dev	N	Mean	St Dev		CL	Upper CL
Rel Liver Wt	8	5.27	0.48	11	5.01	0.44	7	5.07	0.48	0.072058	-0.47	0.61
ALT	8	27.38	6.25	11	19.91	5.45	7	24.00	9.17	-0.35795	-8.29	7.58
AST	8	42.13	6.45	11	40.36	12.42	7	42.14	10.87	-0.89854	-13.10	11.30
SDH	8	24.86	3.30	11	22.54	2.99	7	23.43	4.18	0.27086	-3.71	4.25

0.5:0.5 BDCM:CDBM
Dose = 1.0 mmol/kg/day

End Point	Chem A			Chem B			Mixture			L	Confidence Interval	
	N	Mean	Std Dev	N	Mean	St Dev	N	Mean	St Dev		CL	Upper CL
Rel Liver Wt	7	6.14	0.37	8	6.19	0.42	5	6.57	0.67	-0.40968	-1.06	0.24
ALT	7	91.71	62.56	8	72.75	33.67	5	108.20	104.37	-25.9679	-117.94	66.00
AST	7	81.00	67.15	8	71.25	26.40	5	78.80	54.86	-2.675	-73.10	67.75
SDH	7	70.70	23.73	8	67.13	27.24	5	109.08	113.63	-40.1675	-122.59	42.25

0.5:0.5 BDCM:CDBM
Dose = 3.0 mmol/kg/day

End Point	Chem A			Chem B			Mixture			L	Confidence Interval	
	N	Mean	Std Dev	N	Mean	St Dev	N	Mean	St Dev		CL	Upper CL
Rel Liver Wt	4	8.81	0.38	1	8.74	.	1	7.81	.	0.964414	N/A	N/A
ALT	4	244.25	258.37	1	657.00	.	1	147.00	.	303.625	N/A	N/A
AST	4	243.50	156.92	1	525.00	.	1	129.00	.	255.25	N/A	N/A
SDH	4	383.70	27.46	1	371.70	.	1	104.00	.	273.7	N/A	N/A

[1]All calculations use original untransformed data.

[2]Shaded areas are statistically significant. Overall type I error rate for this study using Scheffé's method is α=0.05.

Table S14 (cont). Prop-RA Untransformed Data Results for Binary Mixture of BDCM and CDBM-rep[1,2]

0.706:0.294 BDCM:CDBM
Dose = 1.0 mmol/kg/day

	Chem A			Chem B			Mixture				Confidence Interval	
End Point	N	Mean	Std Dev	N	Mean	St Dev	N	Mean	St Dev	L	CL	Upper CL
Rel Liver Wt	7	6.14	0.37	8	6.19	0.42	8	6.17	0.35	-0.01415	-0.47	0.44
ALT	7	91.71	62.56	8	72.75	33.67	8	59.38	17.05	26.76379	-22.41	75.94
AST	7	81.00	67.15	8	71.25	26.40	8	55.75	7.40	22.3835	-25.95	70.72
SDH	7	70.70	23.73	8	67.13	27.24	8	63.94	14.44	5.71145	-21.22	32.64

0.706:0.294 BDCM:CDBM
Dose = 3.0 mmol/kg/day

	Chem A			Chem B			Mixture				Confidence Interval	
End Point	N	Mean	Std Dev	N	Mean	St Dev	N	Mean	St Dev	L	CL	Upper CL
Rel Liver Wt	4	8.81	0.38	1	8.74	.	6	8.02	0.64	0.771884	N/A	N/A
ALT	4	244.25	258.37	1	657.00	.	6	346.17	157.91	19.43183	N/A	N/A
AST	4	243.50	156.92	1	525.00	.	6	256.00	96.94	70.261	N/A	N/A
SDH	4	383.70	27.46	1	371.70	.	6	229.92	143.26	150.2553	N/A	N/A

[1]All calculations use original untransformed data.

[2]Shaded areas are statistically significant. Overall type I error rate for this study using Scheffé's method is α=0.05.

Table S15. Prop-RA Untransformed Data Results for Binary Mixture of CHCl3 and CDBM[1,2]

0.5:0.5 CHCl3:CDBM
Dose = 0.1 mmol/kg/day

End Point	Chem A			Chem B			Mixture			L	Confidence Interval	
	N	Mean	Std Dev	N	Mean	St Dev	N	Mean	St Dev		CL	Upper CL
Rel Liver Wt	8	4.50	0.27	8	4.88	0.52	8	4.76	0.89	-0.06814	-0.77	0.63
ALT	8	35.50	26.59	8	25.88	4.29	7	29.86	6.28	0.830357	-18.69	20.35
AST	8	37.50	4.24	8	36.63	7.50	7	49.43	6.92	-12.3661	-19.98	-4.76
SDH	8	18.53	2.64	8	17.85	2.63	7	24.79	9.20	-6.59821	-13.19	-0.01

0.5:0.5 CHCl3:CDBM
Dose = 1.0 mmol/kg/day

End Point	Chem A			Chem B			Mixture			L	Confidence Interval	
	N	Mean	Std Dev	N	Mean	St Dev	N	Mean	St Dev		CL	Upper CL
Rel Liver Wt	8	6.09	0.29	10	6.31	0.43	9	6.01	0.58	0.189974	-0.29	0.67
ALT	8	82.13	49.37	10	67.70	62.65	9	74.11	35.36	0.801389	-53.61	55.21
AST	8	66.88	44.34	10	66.80	55.82	9	93.89	99.11	-27.0514	-102.64	48.54
SDH	8	48.43	27.99	10	51.32	22.07	8	57.93	21.20	-8.0525	-34.54	18.44

0.5:0.5 CHCl3:CDBM
Dose = 3.0 mmol/kg/day

End Point	Chem A			Chem B			Mixture			L	Confidence Interval	
	N	Mean	Std Dev	N	Mean	St Dev	N	Mean	St Dev		CL	Upper CL
Rel Liver Wt	4	6.74	1.26	3	8.05	0.76	5	7.77	1.00	-0.37062	-2.17	1.43
ALT	4	107.75	120.52	3	186.33	74.14	5	544.60	812.77	-397.558	-1336.83	541.71
AST	4	269.25	181.73	3	397.33	130.59	5	698.20	737.64	-364.908	-1234.12	504.30
SDH	4	296.95	199.21	3	265.33	137.20	5	386.74	234.70	-105.598	-456.79	245.60

[1]All calculations use original untransformed data.

[2]Shaded areas are statistically significant. Overall type I error rate for this study using Scheffé's method is α=0.05.

Table S15 (cont). Prop-RA Untransformed Data Results for Binary Mixture of CHCl3 and CDBM[1,2]

0.867:0.133 CHCl3:CDBM
Dose = 1.0 mmol/kg/day

End Point	Chem A			Chem B			Mixture			L	Confidence Interval	
	N	Mean	Std Dev	N	Mean	St Dev	N	Mean	St Dev		CL	Upper CL
Rel Liver Wt	8	6.09	0.29	10	6.31	0.43	8	6.01	0.42	0.110483	-0.37	0.59
ALT	8	82.13	49.37	10	67.70	62.65	8	88.50	93.06	-8.29352	-94.45	77.86
AST	8	66.88	44.34	10	66.80	55.82	8	63.13	42.23	3.740025	-55.98	63.46
SDH	8	48.43	27.99	10	51.32	22.07	8	47.66	29.79	1.147535	-31.35	33.65

0.867:0.133 CHCl3:CDBM
Dose = 3.0 mmol/kg/day

End Point	Chem A			Chem B			Mixture			L	Confidence Interval	
	N	Mean	Std Dev	N	Mean	St Dev	N	Mean	St Dev		CL	Upper CL
Rel Liver Wt	4	6.74	1.26	3	8.05	0.76	5	7.71	0.51	-0.78663	-2.40	0.82
ALT	4	107.75	120.52	3	186.33	74.14	5	159.00	45.74	-40.7984	-193.92	112.32
AST	4	269.25	181.73	3	397.33	130.59	5	211.00	115.23	75.28508	-188.15	338.72
SDH	4	296.95	199.21	3	265.33	137.20	5	255.14	148.92	37.60498	-264.75	339.96

[1]All calculations use original untransformed data.

[2]Shaded areas are statistically significant. Overall type I error rate for this study using Scheffé's method is α=0.05.

Table S16. Prop-RA Untransformed Data Results for Binary Mixture of CDBM and CHBr3[1,2]

0.5:0.5 CDBM and CHBr3
Dose = 0.1 mmol/kg/day

End Point	Chem A			Chem B			Mixture			L	Confidence Interval	
	N	Mean	Std Dev	N	Mean	St Dev	N	Mean	St Dev		CL	Upper CL
Rel Liver Wt	11	5.02	0.39	11	5.08	0.25	8	5.27	0.36	-0.21684	-0.58	0.14
ALT	11	19.64	1.96	11	22.27	5.88	9	19.56	3.64	1.39899	-2.88	5.68
AST	11	46.36	22.67	11	45.45	26.95	9	35.78	6.18	10.13131	-11.65	31.92
SDH	11	13.97	1.70	11	13.86	2.36	9	13.88	1.93	0.040404	-2.03	2.11

0.5:0.5 CDBM and CHBr3
Dose = 1.0 mmol/kg/day

End Point	Chem A			Chem B			Mixture			L	Confidence Interval	
	N	Mean	Std Dev	N	Mean	St Dev	N	Mean	St Dev		CL	Upper CL
Rel Liver Wt	10	6.40	0.24	11	6.43	0.33	7	6.58	0.46	-0.16748	-0.55	0.22
ALT	10	60.60	44.74	11	42.45	30.23	7	36.00	9.61	15.52727	-22.29	53.34
AST	10	64.50	32.56	11	50.00	19.13	7	48.43	6.00	8.821429	-17.49	35.14
SDH	10	28.94	11.00	11	24.41	14.34	7	21.47	4.19	5.203117	-7.75	18.16

0.5:0.5 CDBM and CHBr3
Dose = 3.0 mmol/kg/day

End Point	Chem A			Chem B			Mixture			L	Confidence Interval	
	N	Mean	Std Dev	N	Mean	St Dev	N	Mean	St Dev		CL	Upper CL
Rel Liver Wt	5	7.59	0.46	4	7.55	0.58	5	6.96	0.52	0.611068	-0.20	1.42
ALT	5	705.40	278.77	4	171.75	82.43	5	221.60	191.59	216.975	-111.91	545.86
AST	5	600.60	256.70	4	214.75	81.06	5	225.60	167.87	182.075	-117.27	481.42
SDH	5	143.38	99.96	4	61.08	20.67	5	96.72	101.32	5.5075	-130.97	141.98

[1]All calculations use original untransformed data.

[2]Shaded areas are statistically significant. Overall type I error rate for this study using Scheffé's method is α=0.05.

Table S16 (cont). Prop-RA Untransformed Data Results for Binary Mixture of CDBM and CHBr3[1,2]

0.909:0.091 CDBM:CHBr3
Dose = 1.0 mmol/kg/day

End Point	Chem A			Chem B			Mixture			L	Confidence Interval	
	N	Mean	Std Dev	N	Mean	St Dev	N	Mean	St Dev		CL	Upper CL
Rel Liver Wt	10	6.40	0.24	11	6.43	0.33	9	6.30	0.41	0.100385	-0.28	0.48
ALT	10	60.60	44.74	11	42.45	30.23	9	51.67	37.56	7.282097	-35.82	50.38
AST	10	64.50	32.56	11	50.00	19.13	9	61.22	34.30	1.958278	-31.10	35.01
SDH	10	28.94	11.00	11	24.41	14.34	9	26.31	14.46	2.216576	-13.05	17.48

0.909:0.091 CDBM:CHBr3
Dose = 3.0 mmol/kg/day

End Point	Chem A			Chem B			Mixture			L	Confidence Interval	
	N	Mean	Std Dev	N	Mean	St Dev	N	Mean	St Dev		CL	Upper CL
Rel Liver Wt	5	7.59	0.46	4	7.55	0.58	2	8.52	0.36	-0.93904	-2.15	0.27
ALT	5	705.40	278.77	4	171.75	82.43	2	156.50	36.06	500.3379	2.98	997.70
AST	5	600.60	256.70	4	214.75	81.06	2	133.50	50.20	431.9877	-29.12	893.09
SDH	5	143.38	99.96	4	61.08	20.67	2	61.25	22.42	74.64025	-101.60	250.88

[1]All calculations use original untransformed data.

[2]Shaded areas are statistically significant. Overall type I error rate for this study using Scheffé’s method is α=0.05.

Homogeneity of Variances Test Results for Binary Mixtures For Analysis of Departures from Proportional Response Addition

To test for departures from proportional response addition (Prop-RA), a linear contrast is constructed for a given experiment for each dose/ratio/endpoint combination, and homogeneity of variance (HOV) is assumed across the two single chemical and the binary mixture datasets. To calculate a 95% Scheffé confidence interval for the linear contrast estimate used to analyze departures from Prop-RA, we generate an unbiased estimate of the variance of the linear contrast estimate. This calculation includes a value for the mean square error, which is a pooled weighted average of the three individual dataset variances.

To satisfy the HOV assumption, we used the O'Brien test for HOV. Based on published research, the O'Brien test was chosen for several reasons that are generally characteristic of our THM datasets. The O'Brien test is: the only HOV method that has adequate control of type I error rates for average sample sizes < 10; not sensitive to normality assumptions of the data; and, controls for type I error rate across all population shapes (Wang et al., 2017). In general, it is also not sensitive to skewed data (i.e., data points out in the tail of the distribution as compared with other values in the dataset), although this can sometimes be an issue with small sample sizes.

Supporting HOV results are shown below in Tables S17-S24 and Figures S1-S4. "Table 1" in footnote a refers to Table 1 of the main manuscript. In the figures, the lower and upper ends of the box represent the 1st and 3rd quartiles of the data distribution. Yellow-highlights identify HOV p-values that were higher with the log transform, indicating more homogeneity of variance, and blue-highlights identify HOV p-values that were lower.

HOV testing results were helpful in ascertaining the appropriateness of applying our method for determining departures from Prop-RA to the relative liver weight and serum enzyme data for the THMs. Because HOV testing showed general consistency (nonsignificant differences) across all but 2 experiments for dose/ratio/endpoint combinations for PcLiv, we chose to use the original untransformed data for testing departures from Prop-RA. In general, HOV testing results for the serum enzyme data showed improvements in similarity of variances using the transformed data across all experiments for dose/ratio/endpoint combinations. Evidence included O'Brien test results and visual inspection of changes in the similarities of the variances. Thus, for AST, ALT and SDH, we chose to use the Log10 transformed data for testing departures from Prop-RA.

Table S17. Testing Equality of Variances- Relative Liver Weight

Dose (mmol/ kg-d)	Ratio Studied[a]	Experiment[b,c]							
		1	1A	2	3	4	4A	5	6
0.1	1:1	ns	ns	ns	ns	ns	ns	ns	ns
1.0	1:1	0.01	ns	ns	ns	ns	ns	ns	ns
3.0	1:1	ns	ns	ns	ns	ns	d	ns	ns
1.0	envir.	0.02	ns	ns	ns	ns	ns	ns	ns
3.0	envir.	ns	ns	ns	ns	ns*	e	ns	ns

a. See table 1 for the chemicals and environmentally relevant ratios used in each experiment
b. ns=not statistically significant (p<0.05) for O'Brien variance test. When shown, value is the significance level for that dose-ratio combination.
c. Areas with an * represent data that were statistically different from Prop-RA.
d. No calculation, n=1 for mixture data and for one component's data.
e. ns but n=1 for one component's data.

Table S18. Testing Equality of Variances- Log(Relative Liver Weight)

Dose (mmol/ kg-d)	Ratio Studied[a]	Experiment[b,c]							
		1	1A	2	3	4	4A	5	6
0.1	1:1	ns	ns	ns	ns	ns	ns	ns	ns
1.0	1:1	0.02	ns	ns	ns	ns	ns	ns	ns
3.0	1:1	ns	ns	ns	ns	ns	d	ns	ns
1.0	envir.	0.04	ns	ns	ns	ns	ns	ns	ns
3.0	envir.	ns	ns	ns	ns	ns*	e	ns	ns

a. See table 1 for the chemicals and environmentally relevant ratios used in each experiment
b. ns=not statistically significant (p<0.05) for O'Brien variance test. When shown, value is the significance level for that dose-ratio combination.
c. Areas with an * represent data that were statistically different from Prop-RA.
d. No calculation, n=1 for mixture data and for one component's data.
e. ns but n=1 for one component's data.

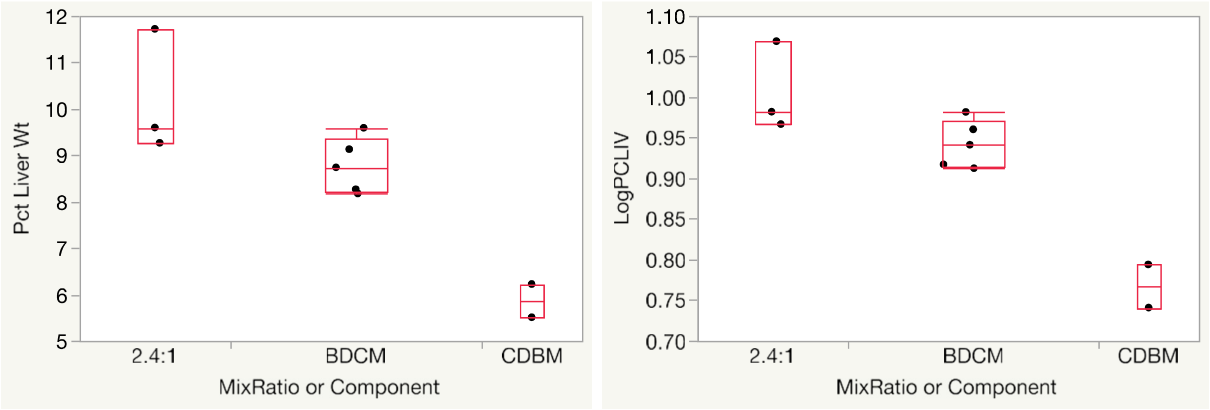

Figure S1. HOV Analysis for (left) percent liver weight or (right) log10(percent liver weight), experiment #4, environmental mix ratio and dose of 3.0 mmol/kg/d

Table S19. Testing Equality of Variances- ALT

Dose (mmol/ kg-d)	Ratio Studied[a]	Experiment[b,c]							
		1	1A	2	3	4	4A	5	6
0.1	1:1	ns	ns	ns	ns	ns	ns	ns	0.01
1.0	1:1	<0.01	ns	ns	ns	ns	ns	ns	ns
3.0	1:1	ns	ns	<0.01	0.03	ns	[d]	ns	ns
1.0	envir.	0.01	0.02	ns	ns	ns	ns	ns	ns
3.0	envir.	ns	ns	ns	ns	ns	[e]	ns	ns*

a. See table 1 for the chemicals and environmentally relevant ratios used in each experiment
b. ns=not statistically significant ($p<0.05$) for O'Brien variance test. When shown, value is the significance level for that dose-ratio combination.
c. Areas with an * represent data that were statistically different from Prop-RA.
d. No calculation, n=1 for mixture data and for one component's data.
e. p=0.04 but n=1 for one component's data.

Table S20. Testing Equality of Variances- log10(ALT)

Dose (mmol/ kg-d)	Ratio Studied[a]	Experiment[b,c]							
		1	1A	2	3	4	4A	5	6
0.1	1:1	ns	ns	ns	ns	ns	ns	ns	0.02
1.0	1:1	0.04	ns	ns	ns	ns	ns	ns	ns
3.0	1:1	ns	ns	0.04*	0.02	ns	[d]	ns	ns
1.0	envir.	ns	ns	ns	ns	ns	ns	ns	ns
3.0	envir.	ns	ns	ns	ns	ns	[e]	0.04	ns*

a. See table 1 for the chemicals and environmentally relevant ratios used in each experiment
b. ns=not statistically significant ($p<0.05$) for O'Brien variance test. When shown, value is the significance level for that dose-ratio combination.
c. Areas with an * represent data that were statistically different from Prop-RA.
d. No calculation, n=1 for mixture data and for one component's data.
e. $p<0.01$ but n=1 for one component's data.

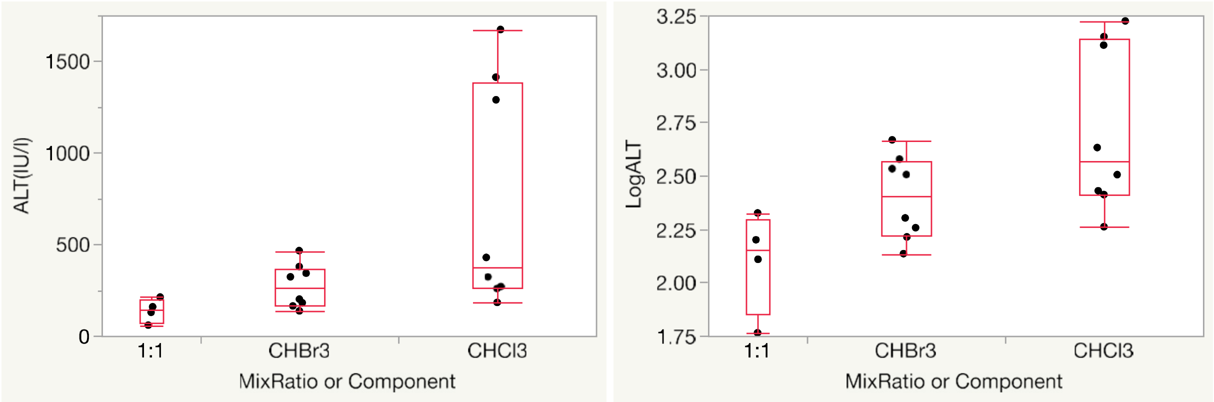

Figure S2. HOV Analysis for (left) ALT and (right) Log(ALT), experiment #2, 1:1 ratio and dose of 3.0 mmol/kg/d

Table S21. Testing Equality of Variances- AST

Dose (mmol/ kg-d)	Ratio Studied[a]	Experiment[bcd]							
		1	1A	2	3	4	4A	5	6
0.1	1:1	ns	ns	ns	ns	ns	ns	ns*	ns
1.0	1:1	<0.01	ns	ns	ns	ns	ns	ns	ns
3.0	1:1	ns	ns	0.01	0.01	ns	d	ns	ns
1.0	envir.	<0.01	0.01	ns	ns	ns	ns	ns	ns
3.0	envir.	ns	ns	ns	ns	ns	e	ns	ns

a. See table 1 for the chemicals and environmentally relevant ratios used in each experiment
b. ns=not statistically significant (p<0.05) for O'Brien variance test. When shown, value is the significance level for that dose-ratio combination.
c. Areas with an * represent data that were statistically different from Prop-RA.
d. No calculation, n=1 for mixture data and for one component's data.
e. ns but n=1 for one component's data.

Table S22. Testing Equality of Variances- log10(AST)

Dose (mmol/ kg-d)	Ratio Studied[a]	Experiment[b,c]							
		1	1A	2	3	4	4A	5	6
0.1	1:1	ns	ns	ns	ns	ns	ns	ns*	ns
1.0	1:1	<0.01	ns	ns	ns	ns	ns	ns	ns
3.0	1:1	ns	ns	<0.01	ns	ns	d	ns	ns
1.0	envir.	<0.01*	0.046	ns	ns	ns	ns	ns	ns
3.0	envir.	ns	0.04	ns	ns	ns	e	ns	ns*

a. See table 1 for the chemicals and environmentally relevant ratios used in each experiment
b. ns=not statistically significant (p<0.05) for O'Brien variance test. When shown, value is the significance level for that dose-ratio combination.
c. Areas with an * represent data that were statistically different from Prop-RA.
d. No calculation, n=1 for mixture data and for one component's data.
e. ns but n=1 for one component's data.

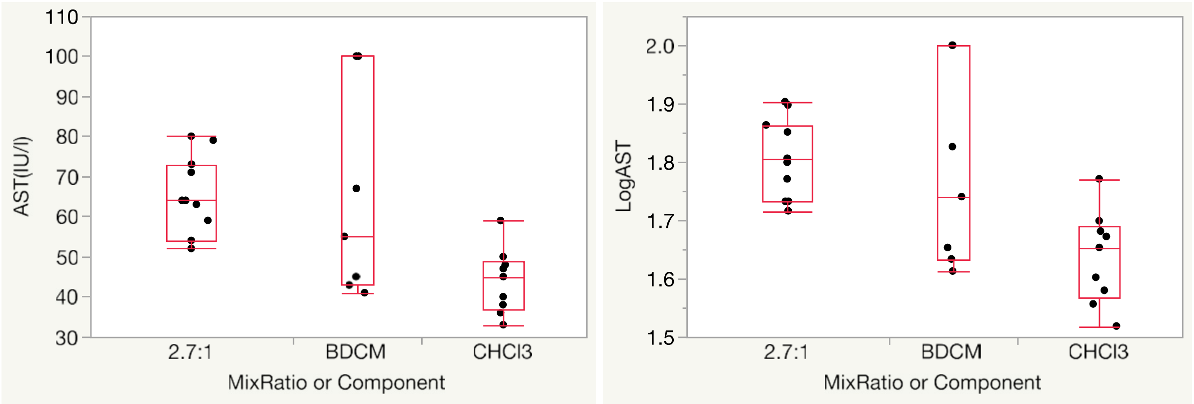

Figure S3. HOV Analysis for (left) AST and (right) Log(AST), experiment #1, environmental mix ratio and dose of 1.0 mmol/kg/d

Table S23. Testing Equality of Variances- SDH

Dose (mmol/ kg-d)	Ratio Studied[a]	Experiment[b,c]							
		1	1A	2	3	4	4A	5	6
0.1	1:1	ns	ns	ns	ns	ns	ns	ns*	ns
1.0	1:1	ns	ns	ns	ns	ns	ns	ns	ns
3.0	1:1	ns	ns	ns	ns	ns	d	ns	ns
1.0	envir.	ns	0.04	ns	ns	ns	ns	ns	ns
3.0	envir.	ns	ns	ns	ns	ns	e	ns	ns

a. See table 1 for the chemicals and environmentally relevant ratios used in each experiment
b. ns=not statistically significant ($p<0.05$) for O'Brien variance test. When shown, value is the significance level for that dose-ratio combination.
c. Areas with an * represent data that were statistically different from Prop-RA.
d. No calculation, n=1 for mixture data and for one component's data.
e. $p<0.01$ but n=1 for one component's data.

Table S24. Testing Equality of Variances- log10(SDH)

Dose (mmol/ kg-d)	Ratio Studied[a]	Experiment[b,c]							
		1	1A	2	3	4	4A	5	6
0.1	1:1	ns	ns	ns	ns	ns	ns	ns	ns
1.0	1:1	ns	ns	ns	0.04	ns	ns	ns	ns
3.0	1:1	ns	ns	0.02	ns*	ns	d	ns	ns
1.0	envir.	ns	ns	ns	ns	ns	ns	ns	ns
3.0	envir.	ns	ns	<0.01	ns	ns	e	ns	ns

a. See table 1 for the chemicals and environmentally relevant ratios used in each experiment
b. ns=not statistically significant ($p<0.05$) for O'Brien variance test. When shown, value is the significance level for that dose-ratio combination.
c. Areas with an * represent data that were statistically different from Prop-RA.
d. No calculation, n=1 for mixture data and for one component's data.
e. $p<0.01$ but n=1 for one component's data.

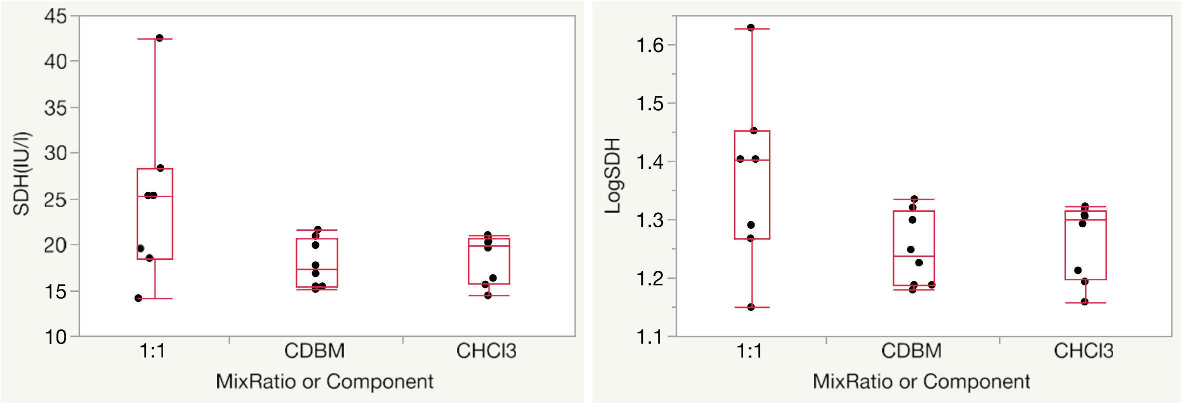

Figure S4. HOV analysis for (left) SDH and (right) Log(SDH), experiment #5, 1:1 mix ratio and dose of 0.1 mmol/kg/d
